# Genome Sequences for *Streptomyces* spp. Isolated from Disease-Suppressive Soils and Long-Term Ecological Research Sites

**DOI:** 10.1128/genomeA.00493-17

**Published:** 2017-06-08

**Authors:** Stephen C. Heinsch, Lindsey Otto-Hanson, Szu-Yi Hsu, Linda Kinkel, Michael J. Smanski

**Affiliations:** a Bioinformatics and Computational Biology, University of Minnesota, Minneapolis, Minnesota, USA; b Department of Plant Pathology, University of Minnesota-Twin Cities, St. Paul, Minnesota, USA; c Department of Biochemistry, Molecular Biology, and Biophysics and BioTechnology Institute, University of Minnesota-Twin Cities, St. Paul, Minnesota, USA

## Abstract

We report here the high-quality genome sequences of three *Streptomyces* spp. isolated as part of a long-term study of microbial soil ecology. *Streptomyces* sp. strain GS93-23 was isolated from naturally disease-suppressive soil (DSS) in Grand Rapids, MN, and *Streptomyces* sp. strains S3-4 and 3211-3 were isolated from experimental plots in the Cedar Creek Ecosystem Science Reserve (CCESR).

## GENOME ANNOUNCEMENT

Disease-suppressive soils (DSSs) have been identified that provide long-lasting and stable protection against numerous bacterial and fungal pathogens (1, 2). In addition to preventing crop loss, DSSs can lower the cost of production by removing the need for pesticide application. However, the evolutionary and molecular mechanisms responsible for the formation and maintenance of DSSs are poorly understood. Recent metagenome sequencing projects (3) and phenotypic characterization of isolated microbial strains (4–7) point to the importance of streptomycetes in the development and maintenance of some DSSs. Streptomycetes are Gram-positive saprophytic soil microbes that are recognized for their ability to produce large numbers of natural compounds with antibiotic and antifungal bioactivities (8). To facilitate the investigation of possible mechanistic roles for streptomycete secondary metabolites in the establishment and maintenance of DSSs, we have begun a genome sequencing campaign for DSS microbial isolates. Here, we report the sequencing and annotation of three high-quality streptomycete genomes from organisms isolated as part of studies into the ecology of DSSs.

Each genome was initially sequenced and assembled from long reads using a Pacific Biosciences single-molecule sequencer at the Mayo Clinic (Rochester, MN). Subread filtering from three single-molecule real-time (SMRT) cells for each genome (two P4-C2 chemistry plus one P6-C4 chemistry) yielded approximately 1 Gb of sequence information for each genome (100× coverage). Contig assembly was performed with HGAP version 3 (9), with default parameters, in the PacBio SMRT Analysis Portal version 2.3. Assemblies were polished using three iterative Quiver processing steps to create consensus sequences with QV scores >60. Remaining indels and single-base errors were corrected using short-read sequences from an Illumina MiSeq at the University of Minnesota Genomics Center (Minneapolis, MN). Paired-end short-read data (2 × 250 bp) with at least 100× coverage were mapped to the long-read assembly reference using Breseq (10), and single-nucleotide polymorphisms (SNPs)/indels were corrected with Pilon (11) to yield high-quality genome sequences. Sequences of large linear chromosomes assembled to a single contig for *Streptomyces* sp. strains GS93-23 (8.24 Mb) and 3211-3 (8.23 Mb) and to two contigs for *Streptomyces* sp. strain S3-4 (7.50 Mb) that could be oriented based on GC skew. Two additional linear plasmids were assembled in both the 3211-3 and S3-4 genomes.

Annotation of the genomes with the Prokka software (12) and antiSMASH 3.0 (13) revealed several dozen gene clusters predicted to encode the biosynthesis of natural products. The numbers and types of protein-coding sequences (7,188, 8,087, and 7,071 for GS93-23, 3211-3, and S3-4, respectively) and biosynthetic gene clusters (26, 38, and 28, respectively) are similar to those seen in other *Streptomyces* genomes. Access to high-quality complete genome sequences for these strains will enable future investigations into the possible roles that the encoded metabolites play in disease suppression.

### Accession number(s).

The sequences have been deposited in GenBank under the following accession numbers: *Streptomyces* sp. GS93-23, CP019457; *Streptomyces* sp. 3211-3, CP020039, with plasmids CP020040 and CP020041; and *Streptomyces* sp. S3-4, CP020042, with plasmids CP020043 and CP020044.
